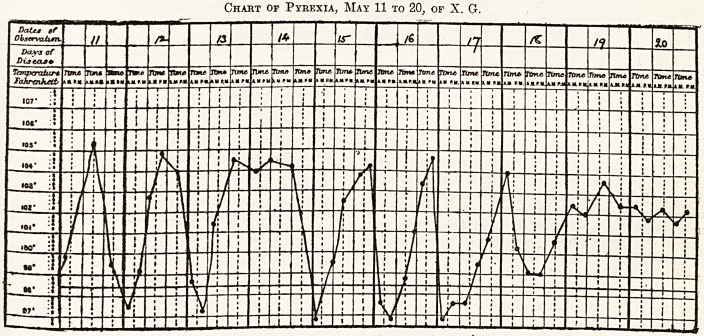# Case Notes of an Obscure Disease

**Published:** 1915-09-25

**Authors:** 


### IN A GENERAL HOSPITAL.

### II. Case Notes of an Obscure Disease.

IN the former article (see THE HOSPITAL, September 18, p. 521) the first case of a series of four was described, and the obscurity of the diagnosis as it presented itself at the time was discussed.

The next case seemed to me to throw light upon the former one, and inclined me to the belief that cerebro-spinal fever could not be so lightly discarded from the differential diagnosis. The patient was a French soldier, X. G., aged 22, a patient in Auxiliary Red Cross Hospital (of France) No. 111, where I saw him and was permitted to take notes of the case by the courtesy of the medical officer in charge, Dr. Nicoletis. On or about March 25 he began to suffer from malaise; a cough started before the end of the month, without any expectoration, but accompanied by slight pain in the right side of the chest. There was at this time no headache, no abdominal pain, no vomiting, no shivering attack. Early in April he improved, and was quite convalescent on April 25. On April 29 he had a shivering fit, and his temperature rose. A copious erythematous rash appeared, chiefly on the limbs, and less profusely on the face and trunk. Every day after this date he had an afternoon or evening shivering fit, with rise of temperature beginning at the same time and reaching a maximum three or four hours later. This was followed by a profuse sweat, with fall of temperature to normal or subnormal. His skin exhaled an odour precisely similar to that of the English soldier whose case has just been described. During the feverish hours there was great malaise, but at other times this was not experienced. Food was well taken; vomiting occurred once. Quinine in massive doses and salicylates were both unable to control or even modify this syndrome. Successive crops of rash appeared every two or three days; all the characters of this rash were the same as in Case 1.

CHART OF PYREXIA, MAY 11 TO 20, OF X. G.

## Figures and Tables

**Figure f1:**